# Does Chronic Tinnitus Alter the Emotional Response Function of the Amygdala?: A Sound-Evoked fMRI Study

**DOI:** 10.3389/fnagi.2017.00031

**Published:** 2017-02-21

**Authors:** Jeff E. Davies, Phillip E. Gander, Deborah A. Hall

**Affiliations:** ^1^Division of Audiology, Faculty of Health and Life Sciences, School of Allied Health Sciences, De Montfort UniversityLeicester, UK; ^2^National Institute for Health Research, Nottingham Hearing Biomedical Research UnitNottingham, UK; ^3^Otology and Hearing Group, Division of Clinical Neuroscience, School of Medicine, University of NottinghamNottingham, UK

**Keywords:** amygdala, double echo, functional magnetic resonance imaging (fMRI), tinnitus, valence, emotion, distress, fMRI

## Abstract

Tinnitus is often associated with strong negative thoughts and emotions which can contribute to a distressing and chronic long-term condition. The amygdala, the “feeling and reacting” part of the brain, may play a key role in this process. Although implicated in several theoretical models of tinnitus, quantification of activity in the human amygdala has only been made possible more recently through neuroimaging methods such as functional magnetic resonance imaging (fMRI) but benefits from modified scanning parameters using a double-echo acquisition for improved BOLD sensitivity. This study thus examined the role of the amygdala in emotional sound processing in people with tinnitus using a novel double-echo imaging sequence for optimal detectability of subcortical activity. Our hypotheses were: (1) emotionally evocative sound clips rated as pleasant or unpleasant would elicit stronger amygdalar activation than sound clips rated as neutral, (2) people with tinnitus have greater amygdalar activation in response to emotionally evocative sounds (relative to neutral sounds) compared to controls.

**Methods:** Twelve participants all with chronic, constant tinnitus took part. We also recruited 11 age and hearing-matched controls. Participants listened to a range of emotionally evocative sound clips; rated as pleasant, unpleasant or neutral. A region-of-interest analysis was chosen to test our a priori hypotheses.

**Results:** Both groups displayed a robust and similar overall response to sounds vs. silence in the following ascending auditory pathways; inferior colliculus, medial geniculate body and the primary auditory cortex. In support of our first hypothesis, the amygdala's response to pleasant and unpleasant sound clips was significantly greater than neutral sounds. Opposing our second hypothesis, we found that the amygdala's overall response to pleasant and unpleasant sounds (compared to neutral sounds) was actually lower in the tinnitus group as compared to the controls.

**Conclusions:** The “muted” amygdala activation observed in the tinnitus group could reflect an internal modification of emotional response perhaps as a result of successful habituation to emotionally negative sound. This interpretation would predict a heightened amygdala emotional response in individuals with a more clinically bothersome tinnitus.

## Introduction

Tinnitus describes the phantom perception of sound, in the absence of an external sound source. Most individuals who experience tinnitus will habituate such that it does not impact on quality of life or emotional state (Davis and El Rafaie, 2000). However, for some the experience of tinnitus can be distressing, affecting sleep, concentration, and mood (Folmer et al., 1999; Cronlein et al., 2016; Tegg-Quinn et al., 2016). Interestingly, the amount of distress attributed to tinnitus is not predicted by its psychoacoustic composition e.g., perceived loudness (Jakes et al., 1985; Leaver et al., 2012). Instead, the emotional evaluation of stimuli appears strongly linked to the limbic system.

The limbic system is a convenient way of describing a number of functionally and anatomically connected brain structures that regulate autonomic and endocrine function, particularly in response to emotional stimuli (Le Doux, 2000). There is no universal agreement on the total list of structures which comprise the limbic system but it includes the cingulate gyrus, parahippocampal gyrus, hippocampal formation, amygdala, septal area, and hypothalamus (Rajmohan and Mohandas, 2007). The limbic system forms the “feeling and reacting brain” but many of the brain areas within the limbic system are also implicated in memory, particularly emotional memory (Rajmohan and Mohandas, 2007).

The amygdala may be particularly significant in its role of mediating emotional responses to sensory stimuli. To decipher specifically how information is relayed between the auditory cortex and amygdala, Kumar et al. (2012) used a range of aversive sound clips in a group of 16 young adults (aged 22–35) with normal hearing. Their analysis focused on the interactions between auditory cortex and amygdala (effective connectivity) using a technique known as dynamic causal modeling. According to the models tested, they concluded the following: unpleasant sound stimuli are first processed and decoded in the auditory cortex before any emotional response can be assigned by the amygdala. Forward connections from the auditory cortex to the amygdala are modulated by acoustic features. The amygdala then modulates the auditory cortex in accordance with the (un)pleasantness of sounds.

Irwin et al. (2011) compared responses to sound clips of real world soundscapes rated as pleasant, neutral or unpleasant to examine the role of auditory and limbic brain networks in normal hearing adults (aged 21–55 years). Irwin et al. (2011) found that highly pleasant or highly unpleasant soundscapes relative to neutral soundscapes evoked greater activation of a number of auditory brain regions including the auditory cortex and the posterior insula, and non-auditory brain regions such as the amygdala. A large response was found to emotionally evocative sounds, irrespective of whether they were pleasant or unpleasant. Irwin described this as a “U-shaped function.” A direct between-hemisphere comparison of amygdala response activation revealed no differences.

The amygdala has been proposed in models of tinnitus to account for the emotional distress that can occur for some individuals in response to “phantom” sounds. Jastreboff's neurophysiological model of tinnitus (1990) includes the amygdala as a key component. Central to this model is the concept that sounds which evoke strong emotional reactions activate limbic and autonomic systems. Sounds can be real physical sounds or phantom sounds such as tinnitus. Typically, repeated exposure to the same sound results in habituation, where the person becomes less aware of that sound. However, when there is an emotional reaction to the sound, any subsequent exposure to the same sound stimulus maintains a conscious awareness of the sound, without habituation. For De Ridder et al. (2011, 2014), a key factor in chronic tinnitus concerns the role of emotional memories. Such memory mechanisms play a role in persistent tinnitus because they result in an extended state of hypervigilance which promotes a sustained state of awareness about the tinnitus. The amygdala is highlighted as a structure of major functional importance because it is not only part of the “distress network” but it also overlaps with brain areas involved in central control of the autonomic system, consistent with Jastreboff's neurophysiological model.

Despite its projected role in tinnitus, the involvement of the amygdala has not been directly measured until more recently (e.g., Shulman et al., 1995; Roy et al., 2009; Crippa et al., 2010; Irwin et al., 2011; Kumar et al., 2012). And even so has been considered as a single functional structure, rather than its known anatomical substructures. Generally these study findings are consistent with the view that the amygdala works with auditory brain regions in the perception of emotionally evocative sounds (both real sounds and phantom sounds). Emotional information (auditory and visual) can be described by two main factors (Bradley and Lang, 2000; Hall et al., 2013). The first factor relates to ratings of pleasantness (valence) and the second factor relates to ratings of vibrancy (arousal). While brain imaging research tends to focus on the coding of pleasantness, for example by measuring the differential response to stimuli that have previously been rated as unpleasant, neutral, or pleasant, Irwin et al. (2011) reported that vibrancy had little effect on the overall brain response.

A majority of the studies examining the response to the emotional dimension of stimulus processing have enrolled young healthy participants (Bradley and Lang, 2000; Costa et al., 2010; Irwin et al., 2011; Kumar et al., 2012). But there are a small number of relevant fMRI studies that have recruited people with tinnitus. Golm et al. (2013) used a non-auditory sentence reading task to stimulate cognitive emotional processing in individuals with varying degrees of tinnitus distress. The task comprised three sentence types, neutral (e.g., regularly I look at my watch), negative (e.g., I often feel sorry for myself), and tinnitus-related (e.g., I will never get rid of the noise). Compared to healthy age- and hearing-matched controls, tinnitus patients showed stronger activations when reading tinnitus-related sentences relative to neutral sentences in many parts of the limbic system: anterior cingulate cortex, mid-cingulate cortex, posterior cingulate cortex, retrosplenial cortex, and insula as well as frontal areas. The tinnitus group were also divided according to levels of perceived tinnitus distress. Individuals with a global score of 31 or higher on the Tinnitus Questionnaire (Hallam, 1996) were assigned into the high distress group. Direct group comparisons (high distress vs. low distress) revealed stronger activity in the left middle frontal gyrus in the high tinnitus distress group, a brain region which Jastreboff (1990) had previously implicated in the integration of sensory and emotional characteristics of tinnitus. Although this study showed some limbic activity, specific amygdala involvement was not found.

Carpenter-Thompson et al. (2014) used emotionally evocative sounds chosen from the International Affective Digital Sounds database (Bradley and Lang, 2007) to directly assess the effects of tinnitus on emotional processing. The authors were motivated by the hypothesis that alterations of the limbic system due to the presence of chronic aversive internal sounds (i.e., tinnitus) are also manifested when processing external affective sounds. They expected to observe an elevated response in the amygdala, parahippocampus, and insula regions of the tinnitus group in response to emotionally evocative sounds, relative to the control groups. They also hypothesized that the tinnitus group would show a heightened response in auditory regions relative to the other two groups. To examine these questions their stimulus set comprised 30 pleasant, 30 unpleasant, and 30 neutral sound clips. In an effort to control for hearing loss, three participant groups were included: “hearing loss with tinnitus” (*n* = 13), “hearing loss without tinnitus,” (*n* = 12) and “normal hearing without tinnitus” (*n* = 12). All groups were gender matched and age matched (mean and SD). Contrary to the author's hypothesis, the tinnitus group did not show an elevated amygdala response in either the pleasant > neutral or unpleasant > neutral contrasts. Direct between-group comparisons failed to show any statistically significant differences in the amygdala response, but the authors highlighted a trend across groups such that “normal hearing without tinnitus” > “hearing loss without tinnitus” > “hearing loss with tinnitus” for the two statistical contrasts described above. And these reached statistical significance only at an uncorrected threshold level of *p* < 0.001 in the normal hearing group for emotionally evocative sounds. The authors suggested two reasons for this: (1) individuals with tinnitus might re-route their emotional signaling pathway to avoid the amygdala and its connections to the auditory cortex, (2) because their participants had mildly bothersome tinnitus, and so may have habituated to the tinnitus. However, we propose a third explanation; it is well known that detecting signal from the amygdala is challenging with fMRI due to its location leading to MR signal loss (Chen et al., 2003; Irwin et al., 2012). It is therefore conceivable that the sensitivity to detecting responses relevant for emotional coding may have been restricted by their choice of fMRI parameters, which were not optimally suited for detection Blood Oxygen Level Dependent (BOLD) contrast in the peri-amygdalar area of the brain.

The present study advances our understanding of how chronic tinnitus impacts upon emotional processing by seeking to conduct an independent confirmation of the previous findings reviewed here. For completeness of reporting, we describe the pattern of sound-related activity in the ascending auditory system, but the focus of this article is on two specific directional hypotheses:

1. Sound clips rated as pleasant and/or unpleasant elicit greater amygdalar activity than neutral sound clips.
2. Compared to age and hearing-matched controls, people with tinnitus have greater amygdalar activity in response to pleasant and unpleasant sounds, relative to neutral sounds.

Our study design sought to overcome several limitations of some of the previous studies. First, we took great care to match groups for age and hearing loss accounting for these known confounding effects (Adjamian et al., 2009). Second, we applied a novel double-echo Echo Planar Imaging (EPI) sequence to improve BOLD sensitivity. This sequence acquires two EPI images per pulse and a sum of these two datasets was created for image analysis (Marciani et al., 2006).

An exploratory research question explored whether differential responses could be detected within the three subdivisions of the amygdala. This was of interest because while previous studies (Golm et al., 2013; Carpenter-Thompson et al., 2014) consider the amygdala as a single homogenous body, this structure can be anatomically delineated into three major subdivisions (Amunts et al., 2005); the laterobasal nuclei (LB), the superficial subnuclei (SF), and the centromedial subnuclei (CM).

## Materials and methods

### Participants

Twelve participants (mean age 65.8 years) with chronic subjective tinnitus and 11 age- and hearing-matched controls (mean age 68.5 years) were recruited through Nottingham Audiology Services or the Ear, Nose and Throat (ENT) department, Queen's Medical Centre, Nottingham. All participants were aged 49–75 years without a history of neurological disorder. See Table 1. for participant demographics and tinnitus characteristics. This study was approved by the National Research Ethics Committee (REC: 09/H0407/8). All participants gave written informed consent prior to taking part. Participants reported in this current study are also described in a previous article (Davies et al., 2014).

**Table 1:** **Group demographics, questionnaire scores and tinnitus characteristics**.

	**No tinnitus group**					**Tinnitus group**					**Tinnitus characteristics**			
	**Sex**	**Age**	**HQ**	**BAI**	**BDI-FS**	**Sex**	**Age**	**HQ**	**BAI**	**BDI-FS**	**Laterality**	**Duration (yrs)**	**THQ**	**TCHQ % annoy**
	F	68	6	2	0	M	72	24	10	4	L	15	25.1	28
	F	71	9	8	3	M	64	14	2	2	L&R	2	35.5	10
	M	58	13	2	0	M	72	14	2	2	L&R	2	60.4	70
	M	68	19	0	3	F	67	8	6	0	L&R	4	61.3	50
	M	75	8	3	0	F	73	11	4	2	IN HEAD	70	21.1	5
	M	68	9	0	0	F	57	18	11	0	IN HEAD	2	63.3	50
	M	60	2	0	0	M	71	22	3	0	IN HEAD	6	68.4	30
	F	75	18	13	4	M	71	11	5	0	L&R	10	32.2	20
	M	66	8	0	0	M	64	11	0	0	L&R	20	30	20
	M	74	2	0	1	F	72	17	3	0	R	13	50.6	35
	M	70	11	14	0	M	49	10	4	2	L&R	2	57.5	50
	~	~	~	~	~	F	58	15	1	1	L&R	40	18.7	25
MEAN	8M/3F	68.5	9.6	3.8	1	7M/5F	65.8	14.6	4.3	1.1	~	15.5	43.7	32.8
*SD*	~	~	5.54	5.34	1.55	~	~	4.9	3.36	1.31	~	20.4	18.32	

*M, male; F, female; L, left; R, right; HEAD, central tinnitus; HQ, hyperacusis questionnaire; BAI, beck anxiety inventory; BDI-FS, beck depression inventory—fast screen; THQ, tinnitus handicap questionnaire; TCHQ, tinnitus case history questionnaire*.

### Clinical profile

**Figure 1:**
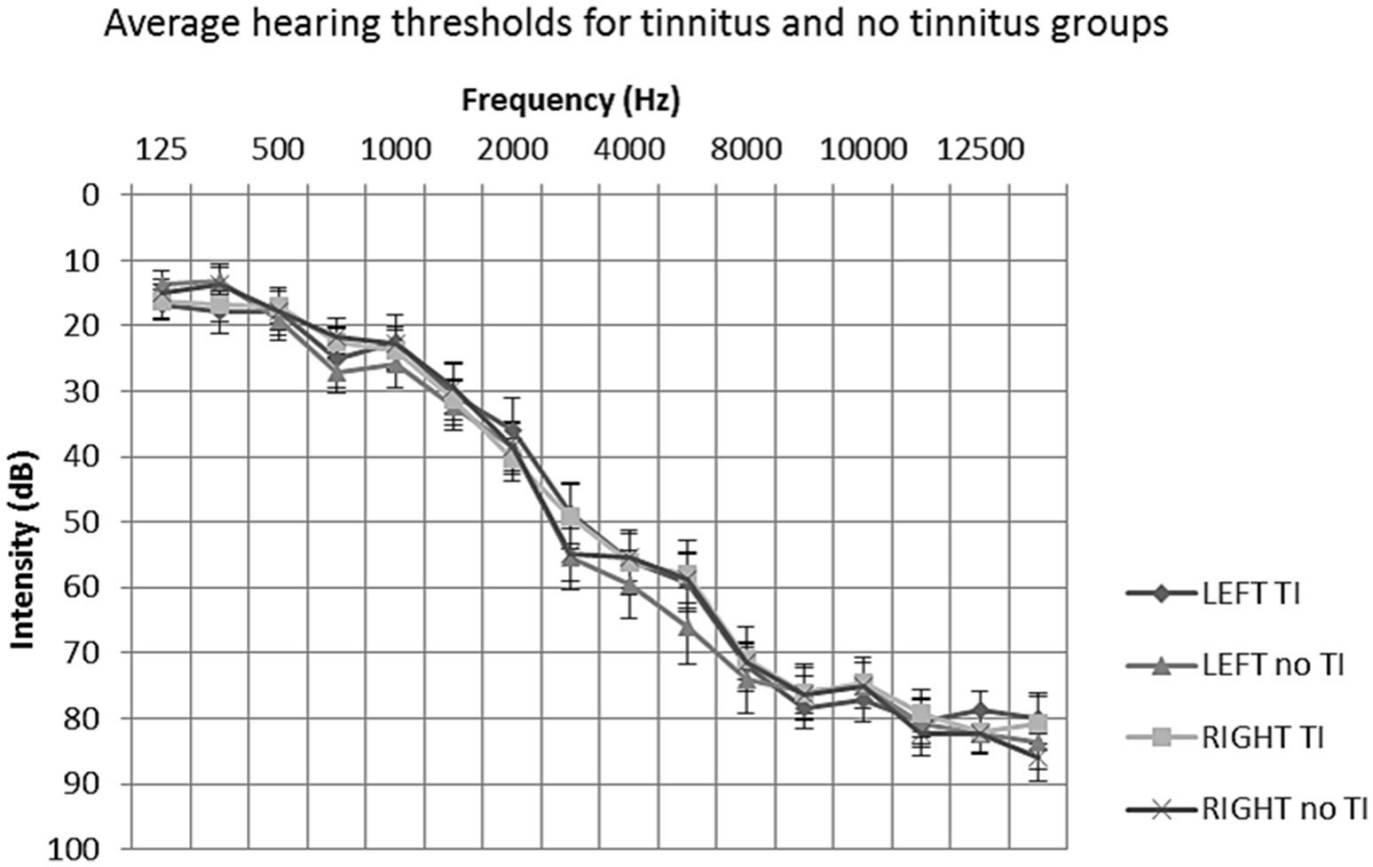
**Mean average audiometric thresholds for tinnitus and no tinnitus groups**. *Post hoc t*-tests of average hearing thresholds found no significant differences between or within participant groups (*P* > 0.05). Error bars represent one standard error of the mean. TI, tinnitus.

All participants underwent extended frequency audiometry (125–14 kHz) prior to scanning. No participants presented with unilateral or asymmetrical hearing loss (as indicated by a between-ear air conduction threshold difference of 15 dB at two or more consecutive frequencies). The average hearing status of both groups could be described as a bilateral, mild to moderately severe sloping sensorineural hearing loss, typical of presbyacusis (see Figure 1). All participants were not considered to have hyperacusis, as indicated by a score of <29 on the hyperacusis questionnaire (Khalfa et al., 2002).

Questionnaire scores are given in Table 1. All participants completed the Hyperacusis Questionnaire (HQ: Khalfa et al., 2002), the Beck Anxiety Inventory (BAI: Beck et al., 1988) and the Beck Depression Inventory—Fast Screen (BDI-FS: Beck et al., 2000). Groups were comparable in terms of clinical profile of co-morbidities. No participant had a HQ score greater than the 28 point cut-off for hyperacusis (Khalfa et al., 2002). All participants had low anxiety, as evidenced by scores <21 on the BAI scale. And all but one participant in each group had a minimal depression, as evidenced by scores ≤ 3 on the BDI-FS scale.

Tinnitus participants also completed the Tinnitus Handicap Questionnaire (THQ: Kuk et al., 1990) and the Tinnitus Case History Questionnaire (TCHQ: Langguth et al., 2007). The tinnitus group had an average global THQ score of 43.7 out of 100 and subjectively rated their tinnitus annoyance at 32.8 out of 100.

### Sound stimuli and task

The present study used 84 sound clips derived from a previously published fMRI study (Irwin et al., 2011). This subset of sound clips were chosen to vary among natural and mechanical real-world sound sources and were previously rated as being pleasant e.g., bird song, unpleasant e.g., car crash or neutral e.g., footsteps. The strategy used to rate the sound clips (see Hall et al., 2013) was as follows; five raters (aged between 21 and 40) rated a total of 219 sound clips using a 9-point visual analog scale with anchor points at either end e.g., 1 = unpleasant/unhappy, 5 = neutral and 9 = pleasant/happy. Scores for each sound clip were comparable across the five raters. In the present study, these sound clips were further reduced down to 84 sound clips (28 pleasant, 28 neutral, and 28 unpleasant) by omitting sounds that were rated either between the anchor end-points (pleasant and unpleasant) or not in the center (neutral) of the 9-point scale. Although our study participants did not rate the sound clips, many of their subjective comments regarding their pleasantness aligned with the formal ratings. Furthermore, we did not expect people with tinnitus to rate sounds differently to that of individuals without tinnitus, for example see Carpenter-Thompson et al. (2014). The intensity of all sound clips was matched at 71 dB A by taking a root-mean-square level average over the 7.8 s clip duration. In an effort to preserve the ecological validity of the listener experience, frequency content was not altered to compensate for participant hearing levels.

**Figure 2:**
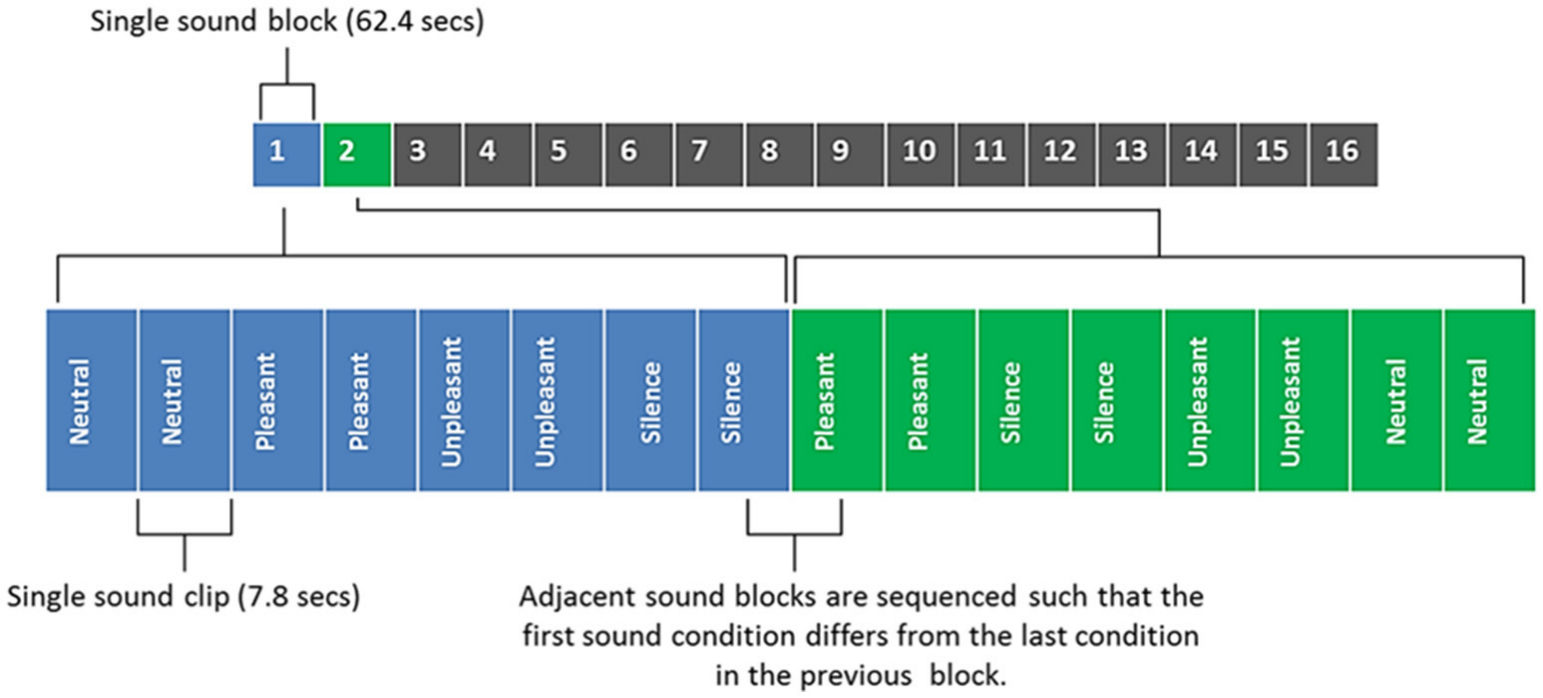
**Sound clip sequence, with blocks 1 and 2 being broken down into their component parts for illustrative purposes**. Each sound block contains 4 different sound conditions/8 sound clips (each sound clip is repeated in quick succession; with a brief inter-stimulus gap of 450 ms). Total duration of all 16 sounds blocks was 16 min 38 s.

Figure 2 shows an illustrative example of the sound clip sequence design which participants had to listen to whilst in the MRI scanner. Each sound clip had a 50 ms onset and offset ramp. Sound clips were presented to participants in a pseudo-randomized order, such that the three categories of sound (neutral, pleasant, and unpleasant) and silence period occurred within a single block that was repeated 16 times. Within each block two segments of sound clips of the same category (or silence) were played in succession (brief inter-stimulus gap of 450 ms) and no repeated sounds were allowed in the two segments. Each participant therefore listened to each sound category (and silent periods) a total of 32 times. The sequence of sounds was constrained to avoid two sequential blocks of the same sound category. Three different unique orderings of sounds were created (i.e., not similar within or across blocks) and randomized across subjects.

### fMRI acquisition

Data were obtained using a Philips Achieva 3.0 Tesla MR scanner (Philips Medical Systems, The Netherlands) and an 8-channel SENSE receiver head coil. For improved BOLD sensitivity in the peri-amygdala area, whole-brain EPI data were acquired by collecting two MR time-series at echo times of 20 and 45 ms after each radio-frequency (RF) pulse (see Marciani et al., 2006). Other EPI parameters were: *TR* = 8250 ms, acquisition time = 2,420 ms, 36 slices, 0 mm slice gap, *FOV* = 240 × 240 mm along the AC-PC line, voxel size = 3 × 3 × 3 mm, acquisition matrix = 80 × 77, 120 volumes, SENSE factor = 2.3, descending slice order). Slice acquisition angle was tilted in line with the axis of the supratemporal plane to capture as much of the brain as possible, with the same negative sloping pitch in the sagittal plane for each subject.

**Figure 3:**
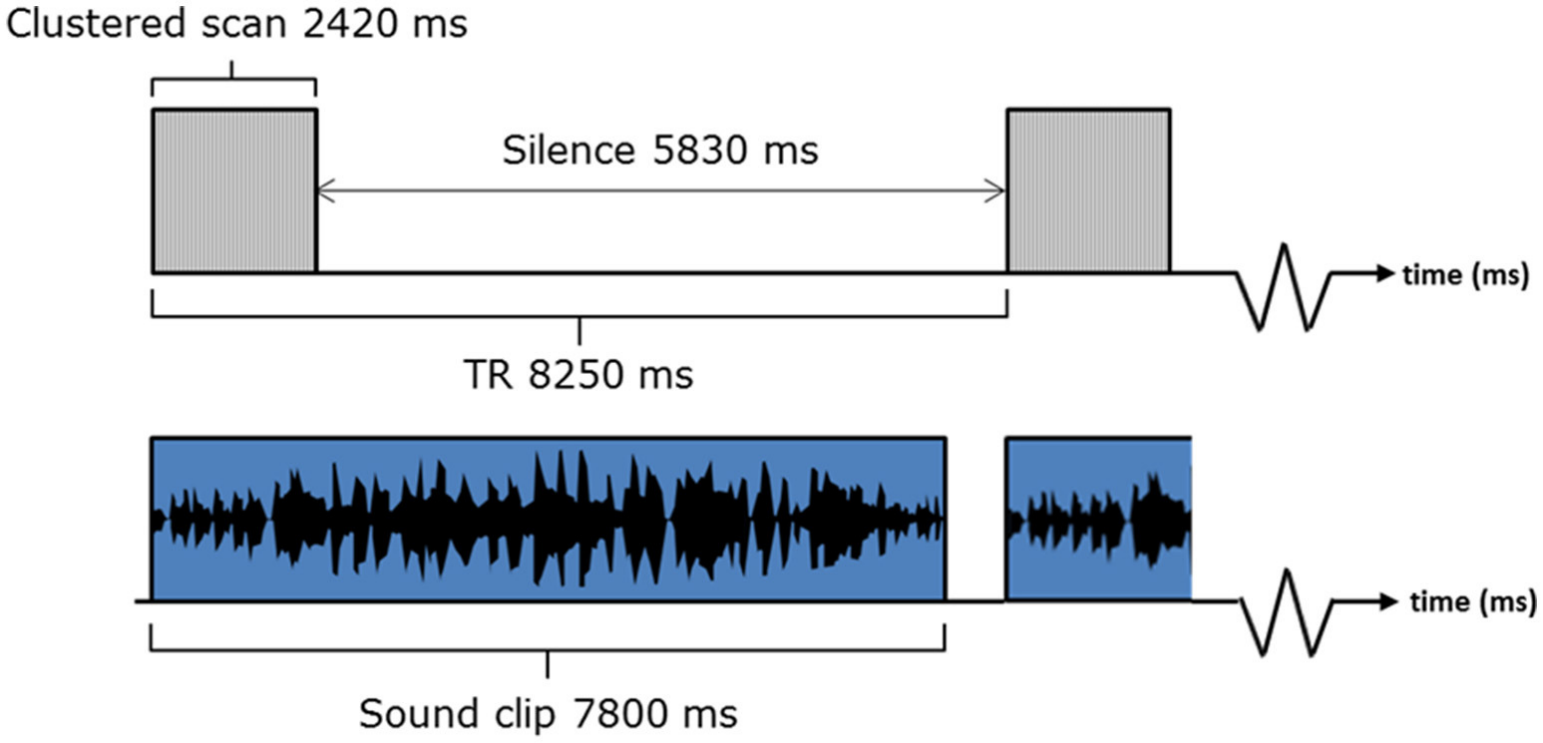
**fMRI listening task paradigm**.

A sparse sampling sequence was adopted, which gave long periods of no scanner noise (5830 ms) in between acquisitions (Hall et al., 1999). Sound clips were presented as shown in Figure 3. Our previous methodological work supports our belief that the small overlap of the sound and the scan acquisition does not affect the BOLD response to the extent that it would affect the statistical contrasts of interest. A 5 min MPRAGE anatomical image was also acquired for each participant (160 slices, *FOV* = 256, voxel size 1 × 1 × 1 mm).

The participants were instructed to keep still with their eyes closed and listen to the sounds. Scanning duration was approximately 16 min. Sound stimuli were delivered through customized circum-aural ear defenders which could provide up to 40 dB attenuation. The ear defenders also employed active noise cancelation, helping to reduce scanner noise by up to an additional 35 dB (Hall et al., 2009). The noise reduction procedures were considered critical for the perception of the sounds, but also to make the scanner environment more suitable for people with tinnitus, whose tinnitus sound could otherwise be masked by the scanner noise or even exacerbated.

### Preprocessing and analysis

First, the two time-series (i.e., at 20 and 45 ms echo times) were combined using a weighted sum; with equal 50/50 weighting using a custom MATLAB script. The single functional time-series was then processed using statistical parametric mapping software SPM8 (http://www.fil.ion.ucl.ac.uk/spm/software/spm8/). Images were realigned, co-registered with the participant's high resolution anatomical scan, normalized to the Montreal Neurological Institute (MNI152) template and spatially smoothed (4 mm full-width at half maximum). A 4 mm smoothing kernel was chosen because of the desire to limit signal spread in small brain regions (Morawetz et al., 2008). The precision of the normalization process was checked visually using the inferior colliculus as a marker since this is easily visible as a discrete structure on both anatomical and functional images.

We adopted a random effects general linear model approach. A first-level fixed-effects analysis was performed on each individual's data, specifying the following t contrasts (sound>silence, pleasant>neutral, unpleasant>neutral, neutral>silence and salient>neutral). Here, “sound” is defined as the sum of all sound conditions. “Salient” is defined as the sum of both pleasant and unpleasant sound conditions such that the contrast “salient>neutral” defines the U-shaped function identified by Irwin et al. (2011). A second-level random effects analysis was then specified to interrogate the hypotheses, with the variance between individuals within a group and between groups accounted in the model.

### Regions of interest

The ascending auditory system comprises the inferior colliculus, medical geniculate nucleus, and auditory cortex in both hemispheres. Localization of activity in the inferior colliculus and medial geniculate body was guided by known co-ordinates (±SEM) for the center of each region. A single inferior colliculus has a volume of about 135 mm^3^ (Kang et al., 2008) which, at our given acquisition resolution, corresponds to 5 voxels, with an MNI co-ordinate center of +/−4, −4, −10 mm. A single medical geniculate nucleus has a volume of about 129 mm^3^ (Kitajima et al., 2015) which corresponds to 5 voxels with a center at ±15, −27, −7 mm.

To test our *a priori* hypotheses relating to sound-evoked activation, analysis targeted pre-defined regions relevant to the hypotheses concerning amygdala in both hemispheres. The average size of the “classic” amygdala is estimated to be 1.24 cm^3^ (*SD* = 0.14), while the average size of the amygdala with wider borders was 1.63 cm^3^ (*SD* = 0.2) (Brabec et al., 2010). At our given acquisition resolution, this corresponded to 23–53 voxels. To localize the amygdala, we used a probabilistic map of the amygdala and its subdivisions based on anatomical data and transformed into MNI space and implemented in the SPM anatomy toolbox v1.8 (Amunts et al., 2005; Eickhoff et al., 2005). This map defined a wide amygdala border as it was thresholded to include those voxels with a <10% probability. While this method reduces the impact of normalization errors on the quantification of overall amygdala activity, it does reduce the precision of segmenting the three amygdalar subdivisions.

## Results

### Sound-related activation in the ascending auditory system

**Figure 4:**
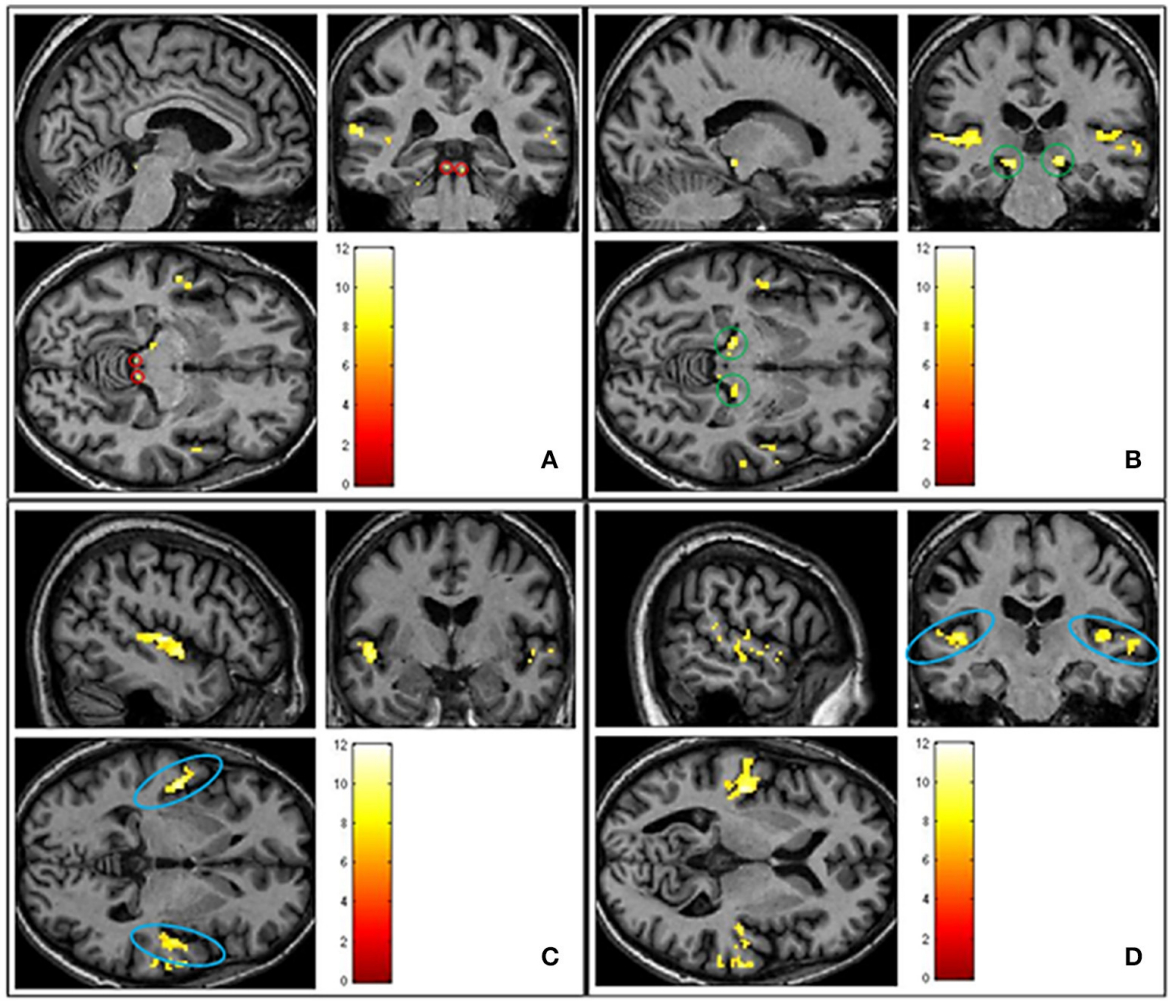
**Shows group averaged (*n* = 23) activation of ascending auditory structures in response to all sound conditions (pleasant, neutral and unpleasant) > silence (*p* < 0.05 FWE corrected)**. **(A)** Inferior colliculus circled in red. **(B)** Medial geniculate body circled in green. **(C)** Primary auditory cortex circled in blue. **(D)** Primary auditory cortex circled in blue.

The first analysis sought to demonstrate sound-related brain activity using a second-level random effects one-sample *t*-test for the contrast “sound>silence” in SPM8 (*n* = 23). Results were whole-brain corrected for family wise error (FWE) and thresholded at *p* < 0.05. We observed robust sound-evoked activation within the inferior colliculus, medial geniculate body and primary auditory cortex, across both hemispheres (see Figure 4). There were no significant differences between groups (*p* > 0.05 FWE corrected).

### Differential sound-evoked activity in amygdala

**Figure 5:**
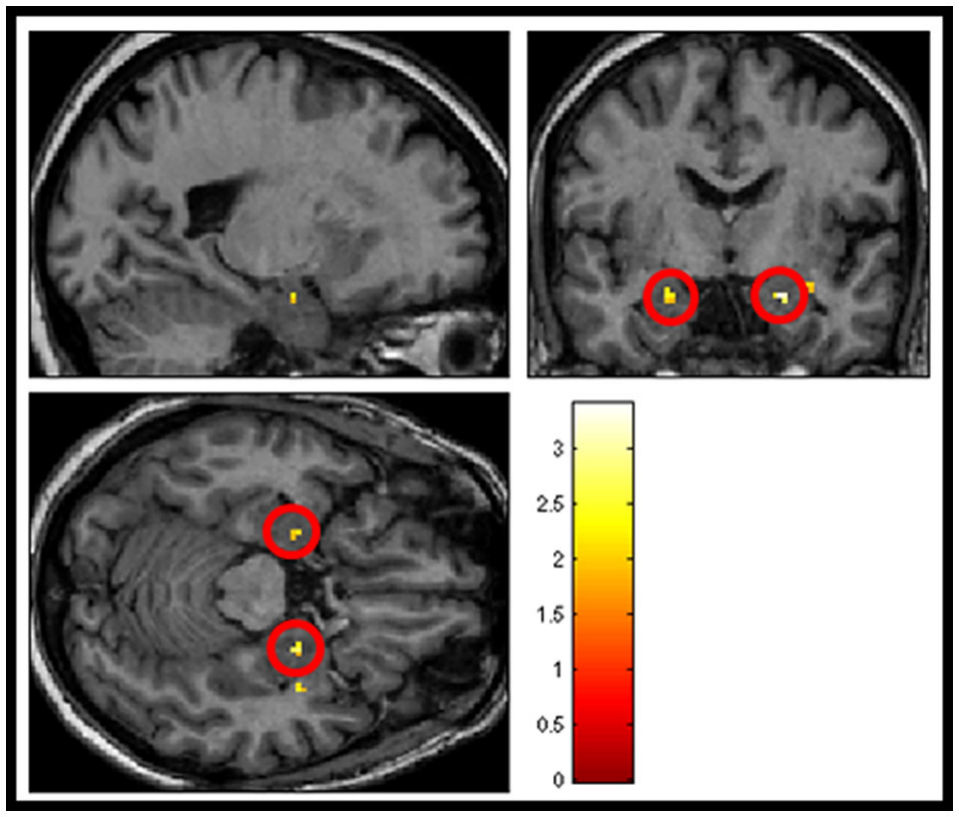
**Shows group averaged (*n* = 23) activation of amygdala structures (circled in red) in response to salience > neutral sound condition, masked with amygdala template (*p* < 0.05 uncorrected)**.

The next analysis addressed the first hypothesis by examining the data using a second-level random effects one-sample *t*-test for the contrast “salient>neutral” in SPM8 (*n* = 23). Because we were testing a specific hypothesis in a pre-defined region of the brain we report uncorrected *p*-values. Within the thresholded borders of the probabilistic map, bilateral amygdala activity was observed to be greater in response to pleasant sounds and unpleasant sounds compared with neutral sounds (*p* < 0.05, uncorrected), potentially consistent with a U-shaped function. The uncorrected result is shown in Figure 5. However, this did not survive statistical thresholding after implementing FWE small volume correction.

### Effects of tinnitus on the response to pleasant and unpleasant sounds

**Figure 6:**
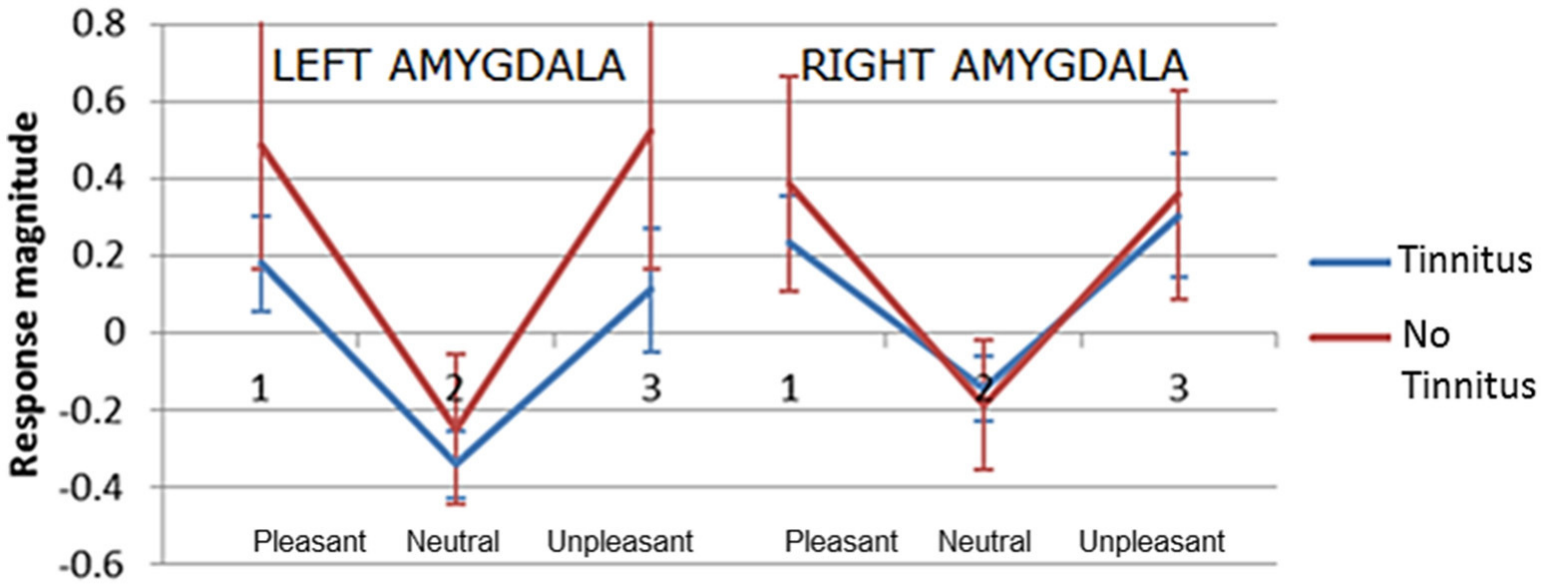
**Plots of mean response magnitude (beta values *p* < 0.05, uncorrected) for the left and right amygdala across three categories of pleasantness (1) pleasant (2) neutral (3) unpleasant**. Error bars represent 95% confidence intervals.

The second hypothesis predicted that people with tinnitus have greater amygdala activity in response to emotionally evocative sounds (relative to neutral sounds) compared to age and hearing-matched controls. Overall amygdala activity was quantified by selecting the peak maxima for the contrast “salient>neutral,” separately in the left and right hemisphere for each individual. Peak maxima fell within the borders of the probabilistic map, as above. For each peak maxima, we extracted the general linear model beta values for each sound condition. These beta values were then submitted to a mixed model ANalysis Of VAriance (ANOVA) in SPSS, with two within-group factors; valence (pleasant, neutral, and unpleasant) and hemisphere (left and right) and one between-group factor (no tinnitus and tinnitus). Results are displayed in Figure 6. Again results in these selected voxels confirmed there was a strong effect of valence [*F*_(2, 42)_ = 102.99, *p* < 0.0001], with the amygdala showing the greatest response to pleasant sounds and unpleasant sounds compared with neutral sounds. However, the response was statistically equivalent in both people with tinnitus and no tinnitus controls [*F*_(1, 21)_ = 3.58, *p* = 0.072] and across hemispheres [*F*_(1, 42)_ = 0.27, *p* = 0.608]. There was no interaction between hemisphere and group [*F*_(1, 42)_ = 2.06, *p* = 0.166].

A significant interaction was observed between valence and group [*F*_(2, 42)_ = 3.63, *p* = 0.035]. *Post-hoc* testing showed that the no tinnitus group had a significantly greater amygdala response to pleasant>neutral sounds (*p* = 0.024) and unpleasant>neutral sounds (*p* = 0.043) compared to the tinnitus group. Direct comparison of the amygdala's response to the neutral>silence condition were not significantly different between groups (*p* = 0.77) suggesting that both groups had a similar “baseline response” to neutral sounds. These main effects and U-shaped pattern were confirmed even when a more conservative ANOVA was re-run to include only those participants with peaks confirmed within the amygdala according to the probabilistic atlas.

### Amygdala subnuclei activation

The voxel-wise statistics and probability value associated with each peak maxima for the contrast “salient>neutral” are presented in Table 2 (tinnitus group) and Table 3 (no tinnitus controls). The assignment of peak voxel to subdivisions of the amygdala provides preliminary exploratory information about the distribution of the greatest response to emotionally evocative sounds (relative to neutral sounds) within this brain region. Whilst all peak voxel amygdala co-ordinates fell within the borders of the probabilistic map, we note that some contiguous voxel clusters extend beyond those borders.

**Table 2:** **Tinnitus group: Amygdala response to the “salient>neutral” contrast (*p* < 0.05 uncorrected)**.

**Subject No**.	**Peak voxel co-ordinates and associated statistics**							**Localization probability**		
	***x***	***y***	***z***	***T*-stat**	***Z*-score**	T2^*^ intensity	**Cluster size**	**LB**	**SF**	**CM**
**LEFT AMYGDALA**										
1	–24	–14	–8	2.19	2.17	35.07	24	20	30	40
6	–26	–6	–30	2.75	2.7	26.95	7	50	0	0
17	–22	–10	–24	3.01	2.94	59.42	36	100	10	0
19	–20	–8	–12	2.41	2.38	29.54	10	80	100	20
24	–22	–6	–16	3.29	3.2	50.14	133	60	50	10
25	–26	2	–28	2.68	2.63	34.64	71	0	0	0
29	–26	–6	–24	1.93	1.91	20.62	2	100	0	0
30	–30	–2	–18	1.63 n.s	1.63 n.s	57.08	4	20	0	0
34	–34	–2	–22	2.4	2.36	40.93	4	0	0	0
45	–16	0	–18	2.94	2.87	55.26	9	10	0	0
54	–32	–6	–32	2.44	2.39	41.64	16	0	0	0
74	–28	–6	–20	3.88	3.72	57.78	134	100	0	0
Average						42.42				
*SD*						13.35				
**RIGHT AMYGDALA**										
1	26	–2	–18	2.4	2.37	55.21	17	10	10	0
6	32	–10	–10	2.55	2.51	55.45	3	60	20	0
17	18	–6	–20	2.44	2.4	65.28	17	0	10	0
19	28	–14	–8	2.83	2.77	43.75	10	40	20	20
24	32	–2	–36	3.41	3.31	37.96	42	10	0	0
25	30	2	–24	2.95	3.88	57.84	43	0	0	0
29	30	4	–32	1.49 n.s	1.49 n.s	0	2	10	0	0
30	28	–2	–18	1.84	1.83	69.45	3	30	10	0
34	30	–4	–22	3.05	2.97	49.25	79	70	0	0
45	24	–4	–12	2.47	2.43	60.07	10	20	60	0
54	20	–10	–12	2.23	2.19	59.98	10	10	80	0
74	20	–8	–12	2.27	2.23	45	15	0	80	0
Average						49.93				
*SD*						18.19				
(*T*-stat of 1.66/*p* = 0.049)										

*Statistical outputs are reported for each individual's peak voxel amygdala co-ordinates (across hemispheres). Amygdala subnuclei: SF, superficial; CM, centromedial; LB, laterobasal. n.s, not significant (p > 0.05)*.

**Table 3:** **No tinnitus group: Amygdala response to the “salient>neutral” contrast (*p* < 0.05 uncorrected)**.

**Subject No**.	**Peak voxel co-ordinates and associated statistics**							**Localization probability**		
	***x***	***y***	***z***	***T*-stat**	***Z*-score**	T2^*^ intensity	**Cluster size**	**LB**	**SF**	**CM**
**LEFT AMYGDALA**										
79	–22	–6	–16	2.6	2.54	41.7	23	60	50	10
80	–22	0	–22	3.18	3.09	137.02	60	20	10	0
81	–20	0	–22	4.07	3.88	31.85	53	10	10	0
82	–22	–8	–20	3.2	3.1	39.82	55	90	30	0
83	–24	–2	–22	3.08	2.99	53.07	50	30	10	0
84	–22	–6	–28	2.5	2.45	38.23	12	50	0	0
85	–26	2	–26	1.8	1.78	48.41	3	0	0	0
86	–20	–4	–10	1.57 n.s	1.57 n.s	30.39	1	0	70	10
88	–22	–6	–14	3	2.92	46.41	24	20	50	20
89	–20	–6	–6	2.89	2.82	22.56	5	10	80	20
90	–26	–6	–28	2.55	2.5	40.54	23	60	0	0
Average						48.14				
*SD*						30.70				
**RIGHT AMYGDALA**										
79	20	–6	–12	2.23	2.2	77.17	9	0	80	0
80	32	–2	–22	2.5	2.45	62.82	17	60	0	0
81	32	0	–20	2.41	2.36	54.44	8	0	0	0
82	36	–4	–32	2.7	2.64	39.77	15	10	0	0
83	28	–16	–8	2.63	2.57	45.23	6	30	20	20
84	24	–4	–30	3.21	3.11	35.56	27	0	0	0
85	28	2	–26	1.8	1.78	37.39	1	0	0	0
86	30	–8	–14	1.88	1.87	35.71	3	80	30	0
88	34	–6	–20	2.62	2.57	52.74	14	80	10	0
89	34	2	–26	2.38	2.34	28.96	14	0	0	0
90	30	–2	–20	2.43	2.39	62.67	10	50	0	0
Average						48.40				
*SD*						14.84				
(*T* stat of 1.66/*p* = 0.049)										

*Statistical outputs are reported for each individual's peak voxel amygdala co-ordinates (across hemispheres). Amygdala subnuclei: SF, superficial; CM, centromedial; LB, laterobasal. n.s, not significant (p > 0.05)*.

Bilateral peak activity occurred in 9 out of 12 (75%) tinnitus participants and 9 out of 11 (81.8%) of the no tinnitus controls. Unilateral peak activity occurred in six participants (3 from each group). Only one participant (subject 85 in the no tinnitus group) showed no amygdala activation in either the left or right hemisphere. Peak activity was most likely to be found in the LB subdivision of the amygdala, and least likely to be found in the CM subdivision of the amygdala. This pattern was true across both hemispheres and both for people with tinnitus and no tinnitus controls.

## Discussion

This study examined how the amygdala responds to emotionally evocative sounds in people with and without chronic tinnitus. Using an experimental protocol adapted from a previously published study (Irwin et al., 2011) we were able to measure activation of auditory brain areas and the amygdala in response to emotionally evocative sounds. The main findings of all analyses are now discussed.

### Sound-related activation of ascending auditory pathways

We found significant sound-related activity in several portions of the ascending auditory pathways including the inferior colliculus, medial geniculate body and the primary auditory cortex, across both brain hemispheres. As expected, this replicates the findings of several earlier sound-evoked studies (Hunter et al., 2010; Husain et al., 2011; Irwin et al., 2011; Carpenter-Thompson et al., 2014). Upon direct statistical comparison between groups, we found no differences in activation amongst auditory brain regions. This finding mimics that of Carpenter-Thompson et al. (2014) study which implemented a similar experimental design and also controlled for hearing loss.

### The effects of pleasantness on amygdala activity

In support of our first hypothesis we found that the amygdala's response to sound was significantly modulated by emotional valence. That is, compared with neutral sound clips, the amygdala's response to pleasant and unpleasant sound clips was significantly enhanced. This overall “U-shaped” response to pleasantness reflects the same amygdala response pattern found by Irwin et al. (2011) in young adults with normal hearing. Also in agreement with Irwin et al.'s (2011) data, we found no main effect of hemisphere, suggesting a lack of amygdala dominance. However, the distinctive amygdala U-shaped response pattern that has been independently replicated across both studies seems to suggest a genuine neurophysiological difference in amygdala function between sound conditions.

Contrary to our second hypothesis, we found no significant main effect of group, indicating that the amygdala's overall response to emotionally evocative sounds was similar between groups. Surprisingly however, a consistent trend for higher activation in response to salient sounds compared with neutral sounds was observed in the “no tinnitus group.” Planned contrasts revealed the specific nature of this relationship. Compared to the “tinnitus group,” the “no tinnitus” group had significantly greater amygdala response magnitude to both pleasant and unpleasant sounds (compared to neutral sounds). By comparing the neutral>silence conditions we determined that there was no difference in baseline activation to non-valent sound between our tinnitus and control groups, discounting the possibility of a ceiling effect caused by a raised baseline response from either group. Our findings are able to build on this uncertainty discussed by Carpenter-Thompson et al. (2014) who did not use a silence condition. In opposition to our original hypothesis, these findings seems to indicate a “muting” of the amygdala response amongst individuals with tinnitus. This finding agrees with Carpenter-Thompson et al. (2014) who observed a decreasing trend in amygdala response activation for NH>HL>TIN groups (note this trend was not statistically significant). Two potential interpretations of this finding include: (1) given the chronic nature of tinnitus, amygdala resources could be automatically be recruited to process the valent tinnitus sound, or (2) people with tinnitus may be actively controlling their emotional response to tinnitus. It may therefore be plausible that in an effort to reduce one's emotional reaction to tinnitus, affected individuals suppress amygdala activation through self-modulation in an effort to divert attention away from the experience of chronic tinnitus. In doing so, this leaves less available “resources” for assignment to other emotional stimuli. Supporting this notion, Domes et al. (2010) found that a group of healthy adults were able to modulate activation of their amygdala up or down by increasing or decreasing their emotional response to affective visual stimuli. Notably, our participants had mild to moderate tinnitus distress and may be better / more successful tinnitus habituators.

### Amygdala subnuclei activation

Amygdala activation was found in the vast majority of participants (*n* = 22/23). This number is considerably higher than Irwin et al. (2011) study from which this experimental protocol was adapted, where only 3/16 participants demonstrated suprathreshold amygdala activity. This large difference in amygdala detectability between studies may reflect our application of a double echo imaging sequence, which is known to provide improved signal contrast across the brain and improved BOLD sensitivity across a range of tissues (Posse et al., 1999; Marciani et al., 2006).

In our exploratory research question which explored whether differential responses could be detected within the three subdivisions of the amygdala, we found that peak activity was most likely to be found in the LB subdivision of the amygdala, and least likely to be found in the CM subdivision of the amygdala. This pattern was consistent across both hemispheres and for both study groups. Within the animal literature, it is well known that the LB nuclei acts as the “gateway” for sensory information to the amygdala, receiving input from both the auditory thalamus and from association areas of the auditory cortex (Bordi and LeDoux, 1992). Support for similar involvement of the LB nuclei when processing emotionally evocative auditory stimuli has been presented in more recent human neuroimaging studies (Ball et al., 2007; Kumar et al., 2012). Kumar et al. (2012) found both the LB and the SF nucleus to encode acoustic features necessary for attributing valence. An earlier study by Ball et al. (2007) also found activation of the LB nuclei but in response to both pleasant and unpleasant sounds. Here, the authors thought this finding may reflect a predominance of auditory inputs to LB subnuclei. In line with this literature, our observed decreasing trend of LB>SF>CM subnuclei activation seems to suggest that the LB nuclei played the most active role in processing the emotional auditory stimuli. However, Ball et al. (2007) discuss an important caveat regarding the choice of voxel resolution. Given that the centers of the different amygdala nuclei are at most 1 cm apart (Mai et al., 1997), subdivision-level investigation of the human amygdala is not optimized at 3 mm^3^ resolution. This finding should therefore be interpreted cautiously.

## Conclusion

To summarize, this study used a double-echo imaging sequence to optimally detect amygdala response patterns to emotionally evocative sounds in people with mild to moderately distressing tinnitus. Our main results show a strong modulatory effect of emotional valence on the amygdala's response. This pattern of activation was reduced in individuals with tinnitus, contrary to our expectations.

By using micro-anatomically defined probabilistic maps, we were able to estimate the origins of amygdala peak level activity. In line with previous research, this found the LB nucleus to be most active when processing emotional auditory stimuli. Based on these findings, the amygdala does appear to provide some useful information which could help in the identification and objectification of tinnitus. However, such activation patterns are, up to now, unlikely to be able to differentiate between the true presence or absence of tinnitus on a single subject level. Future studies targeting amygdala function should carefully consider fMRI parameters to ensure sufficient signal quality from the amygdala regions.

## Author contributions

JD, PG, and DH conceived the study. JD and PG collected the data. JD analyzed the data and wrote the article. PG and DH supervised and provided guidance.

### Conflict of interest statement

The authors declare that the research was conducted in the absence of any commercial or financial relationships that could be construed as a potential conflict of interest.
